# Microcystic adnexal carcinoma of the upper lip misdiagnosed benign desmoplastic trichoepithelioma

**DOI:** 10.1007/s10006-012-0341-x

**Published:** 2012-07-31

**Authors:** Jan Rustemeyer, Stefan Zwerger, Matthies Pörksen, Klaus Junker

**Affiliations:** 1 Department of Oral and Maxillofacial Surgery, Klinikum Bremen–Mitte, School of Medicine of the University of Göttingen, Bremen, Germany; 2 Institute of Pathology, Klinikum Bremen–Mitte, School of Medicine of the University of Göttingen, Bremen, Germany

**Keywords:** Desmoplastic trichoepithelioma, Microcystic adnexal carcinoma, Abbé flap, Upper lip reconstruction

## Abstract

**Background:**

Desmoplastic trichoepithelioma (DT) is a benign appendageal tumour predominately localized on the facial skin. The histological diagnosis can be difficult in some cases. Partial malignant transformation of a DT is a rarity and a complete transformation has never been described in literature.

**Case report:**

A DT of the upper lip was diagnosed histologically by a small biopsy 4 years previously. At presentation, the tumour had enlarged and had partly infiltrated the left side of the upper lip and subnasal region. Histological evaluation confirmed a microcystic adnexal carcinoma but without evidence of malignant transformation of the DT. It appeared that a too-small initial biopsy had led to the incorrect histological diagnosis of a benign tumour. Thus, it was necessary to perform a tumour resection and reconstruction using a two-flap technique including a rotation flap and an Abbé flap. Functional and aesthetic outcomes were good after 6 months. There were no recurrences during a 12-month follow-up.

**Conclusion:**

A facial DT should be resected completely. Patients should be attended for follow-ups, keeping in mind the difficulty of making a proper histological diagnosis from small biopsies or excisions and the consequences of ablative facial surgery. However, in particular cases, subtotal defects of the upper lip region are amenable to reconstruction without gross functional or aesthetic deficits.

## Introduction

Desmoplastic trichoepitheliomas (DTs) amount to less than 1 % of all cutaneous tumours and belong to one entity of the various spectra of benign follicular differentiated appendageal neoplasms of the skin [1]. DTs are predominantly localized in the facial region, with over 80 % affecting females and 70 % involving ages 30 to 60 [2]. In contrast, there are some malignant appendageal tumours, including tumours with follicular differentiation (e.g. pilomatrical carcinoma) and with apocrine and eccrine differentiation (e.g. malignant mixed tumour, microcycstic adnexal carcinoma), that have been well established as entities up to now [3]. A partly malignant transformation of DT seems to be rare, but possible, in cases of multiple familial trichoepithelioma (Brooke–Spiegler syndrome) [4–6]. Malignant transformation of the complete DT has not yet been described. However, we present a case of a microcystic adnexal carcinoma (MAC) in the upper lip region found 4 years after the histological diagnosis of DT.

## Case report

**Fig. 1:**
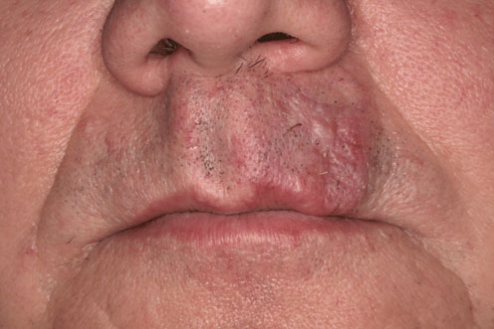
Nodular and plaquelike growth pattern of a microcystic adnexal carcinoma on the left of the upper lip and subnasal region

**Fig. 2:**
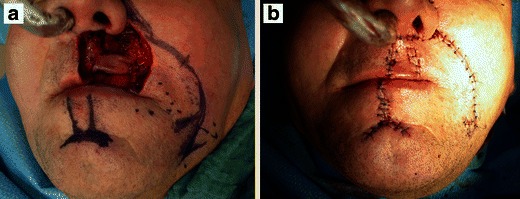
Intraoperative views of the deep-seated resection defect that included the philtrum and upper lip. Incision lines for reconstruction with rotation flap and Abbé flap from the lower lip were marked with blue ink (**a**). Reconstruction of the upper lip, philtrum, and vermillion border with rotation flap and Abbé flap (**b**)

**Fig. 3:**
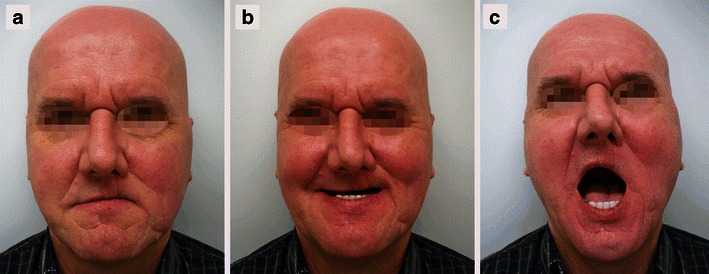
Postoperative views. Lips in repose with competent lip closure (**a**); smiling was nearly symmetrical (**b**); mouth opening was only slightly impaired (**c**)

A 56-year-old Caucasian man had been admitted to our clinic 4 years previously with a 1.2 × 0.5-cm nodular efflorescence of the left side of the upper lip. Biopsy and histological examination provided the diagnosis of a DT. The patient refused complete excision and follow-ups. Four years after initial diagnosis, the patient observed a rapid enlargement of the tumour. At presentation, a 2.8 × 2.4-cm, nodular, plaquelike tumour was found that had palpatorically infiltrated the upper lip and subnasal region (Fig. 1). Deep biopsy now led to the histological diagnosis of MAC. Staging work-up, including MRI of head and neck, thoracic, abdominal, and pelvic CT scans revealed no evidence of local or systemic metastasis. Complete resection of the carcinoma with tumour-free margins of 5 mm resulted in a subtotal defect of the upper lip and philtrum. Immediate reconstruction was performed using a rotation flap from the cheek and lower lip in combination with an Abbé flap for philtrum and vermilion reconstruction (Fig. 2). Six weeks postsurgery and after conditioning, the Abbé flap was separated from its pedicle. Six months after surgery, reconstruction of the defect showed good function and aesthetics (Fig. 3). No recurrences appeared during a 12-month follow-up.

## Discussion

Previous case studies have reported the possibility of malignant transformation of a DT. In these cases, histopathology revealed a transformation zone between the DT and the malignant tumour mass as evidence of partial transformation [6]. In the present case, such transformation zone could not have been obtained histologically after complete resection of the tumour. Nevertheless, taking only this feature into account, a complete malignant overgrowth of a DT could not be definitely ruled out.

**Fig. 4:**
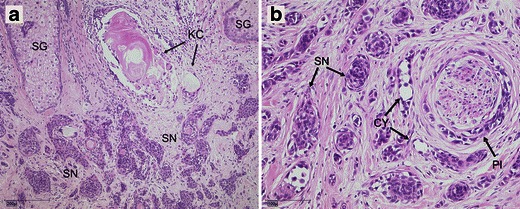
**a** Findings in superficial biopsy of tumour. Small nests (*SN*) and cords of basaloid cells showing a trabecular growth pattern. Several keratin-filled cysts (*KC*) and sebaceous glands (*SG*). No conclusive evidence of malignancy. Findings led to the misdiagnosis of a desmoplastic trichoepithelioma. H&E staining; magnification ×100. **b** Findings in deeper slices after complete tumour excision. Small, solid nests (*SN*) and cystic structures (*CY*). Growth pattern comparable to findings in (**a**). But now evidence of malignancy indicated by infiltration of nerves and perineural spaces (*PI*). Findings led to the diagnosis of a microcystic adnexal carcinoma. H&E staining; magnification ×200

**Fig. 5:**
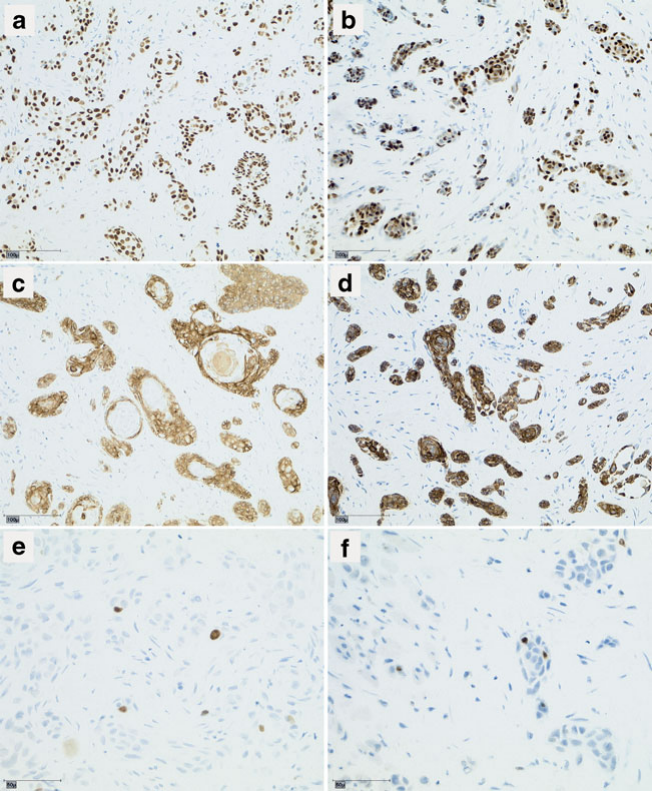
Comparison of immunohistological staining of slices from initial biopsy (left row: (**a**, **c**, **e**) and deeper slices after complete tumour excision (*right row*: (**b**, **d**, **f**)). No different findings in staining. **a** and **b** p63 (nuclear staining); **c** and **d** CK 5/6 (cytoplasmic staining); **e** and **f** Ki67 (staining for proliferating cells). **a**–**d** Magnification ×200; **e** and **f** Magnification ×400

However, the reevaluation of specimens of the initial biopsy by pathologist conceded the case of misdiagnosis by comparing the growth pattern with that found in specimens obtained from the complete tumour excision. This was the most decisive point since infiltrative growth could only be found in deeper slices from the complete tumour specimen (Fig. 4). The immunohistological staining showed no differences between the specimens of the initial biopsy and deeper slices. All specimens revealed negative results for monoclonal antibody staining with BerEP4, staining for epithelial membrane antigen, carcinoembryonic antigen, and cytokeratin (CK) 7 and 20. Positive results could be found for tumour protein p63, nuclear antigen Ki67 (weak positive), and for CK 5/6 (Fig. 5). Hence, malignancy could neither been confirmed nor ruled out by the performed immunohistological devices. Retrospectively and supported by literature [7, 8], the initial incorrect histological diagnosis might be mostly attributed to the fact that the infiltrative growth pattern of MAC could only been distinctively seen in adequate large and deep excisions.

However, a total resection of a MAC with clear surgical margins must be performed. Large defects following tumour resection in aesthetically and functionally sensitive areas, especially in the lip region, are still a challenge for reconstructive surgery, and they are discussed controversially in the literature. Reconstruction of a large defect of the upper lip can be performed by a variety of local flaps or by microvascular free-tissue transfer. In the present case, two local flaps for reconstruction of the upper lip and paranasal defects were performed during one operation. We were able to close the gross soft tissue defect with a rotation flap from the lower lip and cheek. A modified Abbé flap [9] was used to reconstruct the aesthetic units of the vermilion border and the philtrum. These procedures resulted in good aesthetic and functional outcomes in only 6 months. Deglutition and articulation were not affected, and microstomia was avoided. This was particularly important in the present case because the patient wore a full upper denture. Total or subtotal upper lip reconstruction by means of thin, folded fasciocutaneous free flaps also produce good functional and aesthetically acceptable results that avoid additional scars on the lower lip and cheek. However, a disadvantage of this method is the remaining incompetence of the orbicularis oris muscle [10]. The option of a free-tissue transfer becomes especially attractive when, in addition to the lip, there is associated loss of other aesthetic units or bone loss [11]. Accordingly, the decision for using the technique presented in this case was based on the dimension of the defect ruling out the applicability of a free flap for reconstruction and our own experiences and reports in the literature that maintaining the local perioral tissue is more likely to preserve the dynamic and sensory function of the lip than could free flaps [10, 11].
